# Mucinous cystic neoplasm of the liver with polypoid nodule prolapsing into the bile duct: a case report and review of literature

**DOI:** 10.1186/s40792-022-01511-9

**Published:** 2022-09-23

**Authors:** Yasuhiro Fukui, Akihiro Murata, Sadatoshi Shimizu, Kayo Sai, Takuma Okada, Tetsuzo Tashima, Shintaro Kodai, Akishige Kanazawa, Takahiro Okuno

**Affiliations:** 1 Department of Hepato-Biliary-Pancreatic Surgery, Osaka City General Hospital, 2-13-22 Miyakojima-hondori Miyakojima-ku, Osaka City, Osaka 534-0021 Japan; 2 Department of Diagnostic Pathology, Osaka City General Hospital, 2-13-22 Miyakojima-hondori Miyakojima-ku, Osaka City, Osaka 534-0021 Japan

**Keywords:** Mucinous cystic neoplasm of the liver, Growth, Bile duct, Ovarian-like stroma

## Abstract

**Background:**

Mucinous cystic neoplasm of the liver (MCN-L) is a rare cystic tumor as defined by the 2010 World Health Organization classification. MCN-L usually does not communicate with or grow into the bile duct. Herein, we present a rare case of MCN-L with a polypoid nodule protruding into the bile duct.

**Case presentation:**

A 69-year-old woman was referred to our hospital for elevated serum liver enzyme levels and obstructive jaundice. The patient also complained of abdominal pain in the right hypochondriac region. Abdominal ultrasonography showed a cystic lesion in segment 4 (S4) of the liver. Computed tomography revealed a 4-cm multilocular cystic lesion with a thick wall and multiple septal formations, showing a cyst-in-cyst appearance in S4. Endoscopic retrograde cholangiography showed a contrast defect between the left hepatic duct and the common bile duct, which was suspected to be a nodular lesion in the bile duct. Bile cytology and biopsy of the nodular lesion showed no malignant findings. Based on these findings, the differential diagnosis in this patient included intraductal papillary neoplasm of the bile duct and MCN-L, which had malignant potential. The patient underwent left hemihepatectomy, including caudate lobe excision with bile duct resection and right hepatocholangiojejunostomy. Macroscopic findings showed a 40 × 29 mm multilocular cystic lesion with a polypoid nodule that protruded into the left intrahepatic bile duct. As an ovarian-like stroma was observed in both cystic and polypoid lesions microscopically, the histopathological diagnosis was MCN-L. The postoperative course was uneventful, and the patient was discharged 24 days after surgery. The patient is currently alive without recurrence 22 months after the surgery.

**Conclusion:**

Although MCN-L rarely communicates with the bile duct, it is necessary to consider that MCN-L could grow into the bile duct, occasionally causing obstructive jaundice.

## Background

Mucinous cystic neoplasm of the liver (MCN-L) is a rare cystic tumor as defined by the 2010 World Health Organization (WHO) classification [1]. In general, MCN-L occurs most often in the left lobe of the liver of middle-aged women [2]. Histologically, MCN-L is distinguished from intraductal papillary mucinous neoplasm of the bile duct (IPNB) by the presence of ovarian-like stroma (OLS), but it is sometimes difficult to differentiate on imaging. MCN-L rarely communicates with the bile duct, unlike IPNB. Herein we report a rare case of MCN-L that communicated with bile duct and a polypoid nodule prolapsing into the bile duct.

## Case presentation

**Fig. 1:**
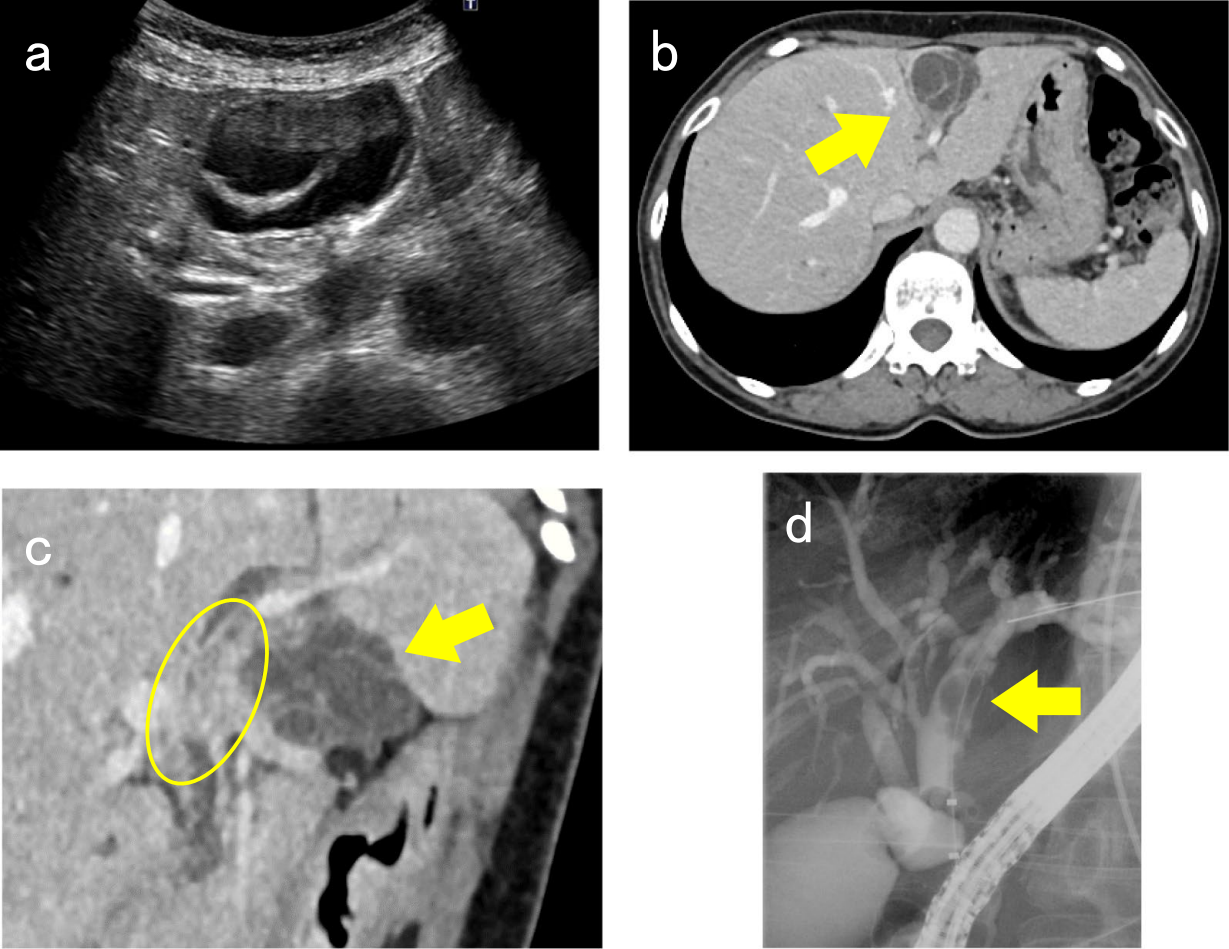
Imaging examination findings. **a** Abdominal ultrasonography showed a 49 × 29-mm cystic lesion with a septum in Segment 4 (S4). **b** Computed tomography (CT) revealed a 4-cm multilocular cystic lesion with multiple septal formations showing a cyst-in-cyst appearance in S4 (arrow). **c** CT revealed polypoid nodule (oval) protruded in the left hepatic duct from the cystic lesion (arrow). **d** Endoscopic retrograde cholangiography showed a contrast defect in the left hepatic duct to common bile duct which was suspected to be a nodular lesion in the bile duct (arrow)

**Fig. 2:**
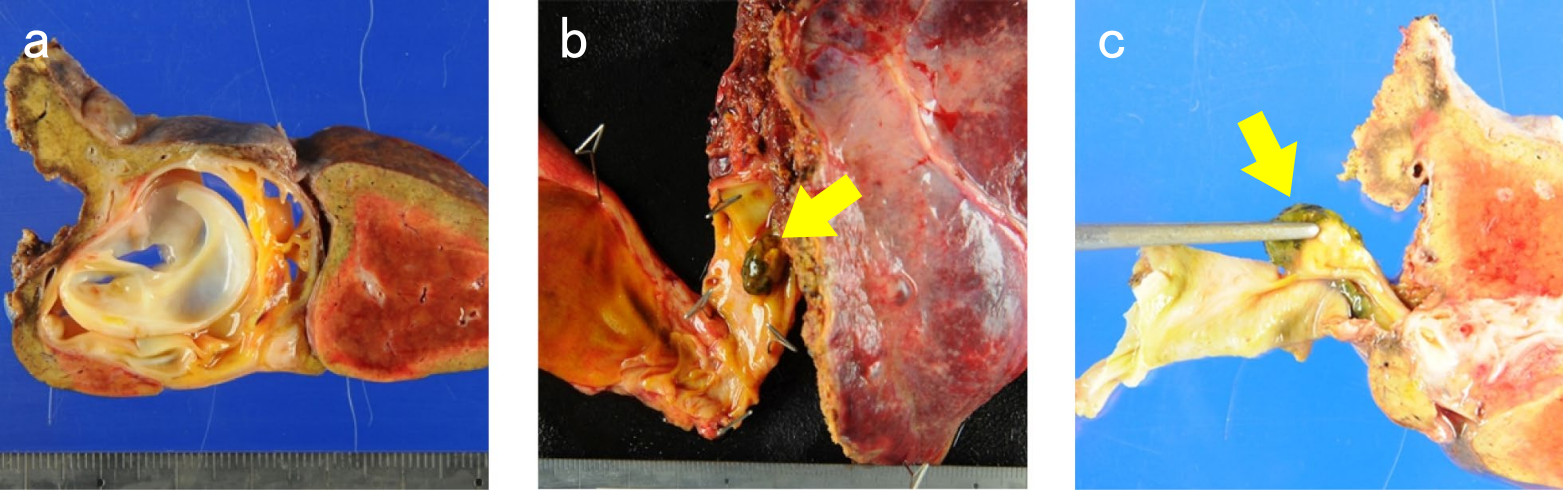
Macroscopic findings of the resected tumor. **a** Macroscopic findings showed a 40 × 29-mm multilocular cystic lesion. **b**, **c** Cystic lesion protruding into the left intrahepatic bile duct, and forming a polypoid lesion (arrow). The polypoid lesion was derived from cystic wall

**Fig. 3:**
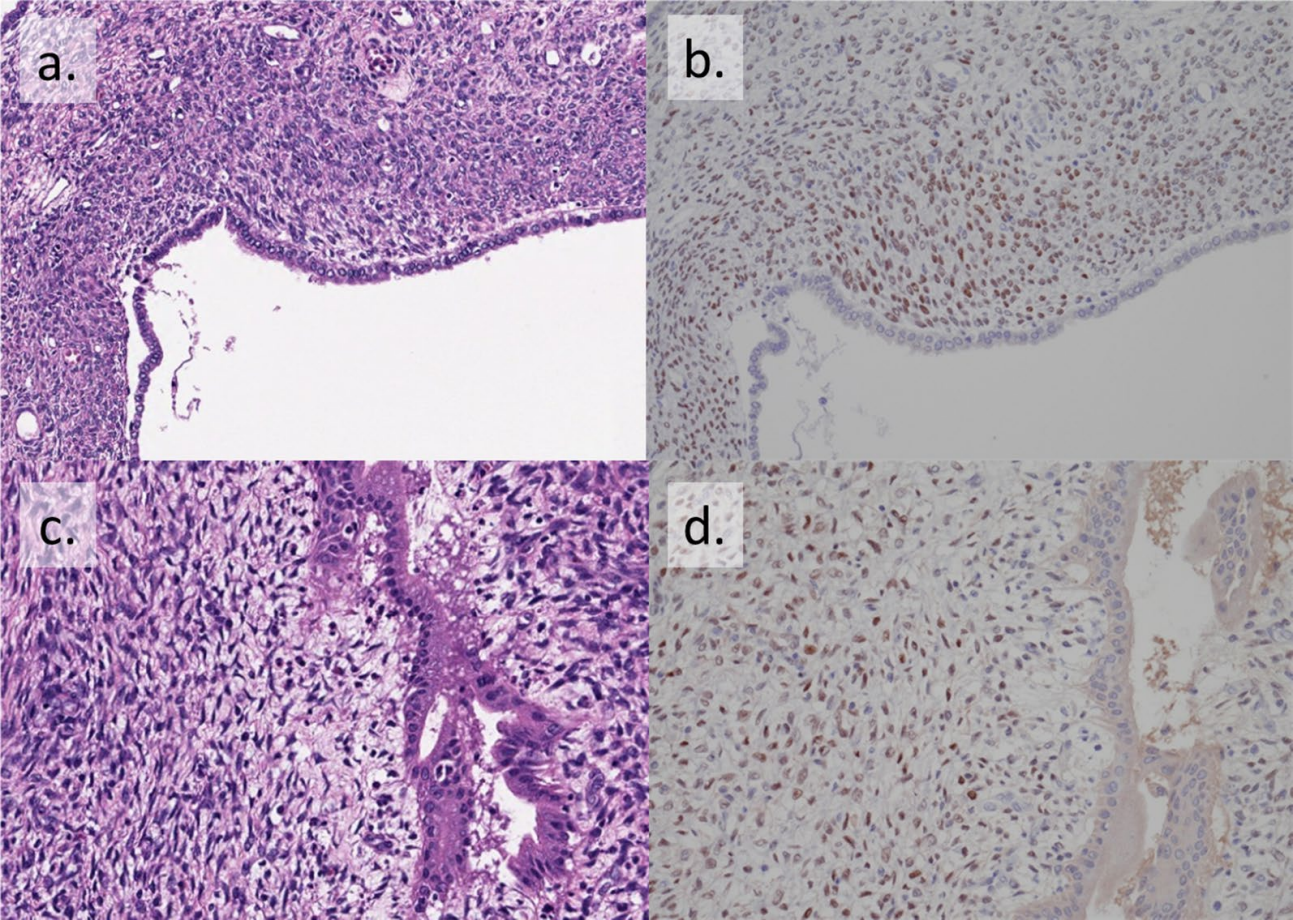
Microscopic findings of the resected tumor. **a** The cyst wall was covered with columnar epithelium, and ovarian-like stroma (OLS) was observed extensively in the cyst wall stroma. **b** Immunostaining showed the sample was positive for estrogen receptors (ER). **c** In the polypoid lesion, OLS was observed as well as cyst wall. **d** Immunostaining also showed that the polypoid lesion was ER positive

A 69-year-old woman was referred to our hospital for the investigation and treatment of elevated serum liver enzyme levels and obstructive jaundice. She also complained of tenderness in her right hypochondriac region. Laboratory tests revealed elevated levels of hepatobiliary enzymes (aspartate transaminase: 91 U/l, alanine transaminase: 249 U/l, alkaline phosphatase: 990 U/l, γ-glutamyl transpeptidase: 761 U/l, total bilirubin: 1.7 mg/dl) and jaundice. Carcinoembryonic antigen, carbohydrate antigen 19-9, alpha fetoprotein, and protein levels induced by vitamin K absence or antagonist-II were within the normal range. The preoperative liver function test was classified as Child–Pugh grade A. Abdominal ultrasonography showed a cystic lesion in segment 4 (S4) of the liver (Fig. 1a). Computed tomography (CT) revealed a 4 cm multilocular cystic lesion with multiple septal formations showing a cyst-in-cyst appearance in S4 (Fig. 1b). In addition, a nodule extending from the left hepatic duct to the common bile duct was observed, which was suspected to have arisen from the cystic wall (Fig. 1c). Endoscopic retrograde cholangiography (ERC) showed a contrast defect from the left hepatic duct to the common bile duct, which was suspected to be a nodular lesion in the bile duct (Fig. 1d). Bile cytology and biopsy of the nodular lesion showed no malignant findings. Based on these findings, the differential diagnosis in this patient included IPNB and MCN-L which had malignant potential. In addition, because the tumor had a nodular part, the coexistence of a malignant neoplasm was suspected. The patient underwent left hemihepatectomy, including caudate lobe excision with bile duct resection and right hepatocholangiojejunostomy. The operative time was 574 min, and blood loss was 490 ml. Macroscopic findings of the resected tumor showed a 40 × 29 mm multilocular cystic lesion with a polypoid nodule prolapsing into the left intrahepatic bile duct and, confirming communication between the tumor and the bile duct (Fig. 2). Microscopic findings revealed that the cyst wall was covered with columnar epithelium and OLS was observed extensively in the cyst wall stroma (Fig. 3a). Immunostaining showed that the cyst wall was positive for estrogen receptors (ER) (Fig. 3b). OLS was also observed in the polypoid lesion (Fig. 3c), and ER was positive in the polypoid lesion (Fig. 3d) as well as cyst wall. Histopathological diagnosis was MCN-L and no malignancy was observed. The postoperative course was uneventful and the patient was discharged 24 days after surgery. Follow-up imaging showed no evidence of recurrent disease 22 months postoperatively.

## Discussion

The WHO classified mucin-producing bile duct tumors of the liver as MCN-L or IPNB in 2010 [1]. MCN-L is defined as a cyst-forming epithelial neoplasm, usually demonstrating no connection with the bile ducts, whereas IPNB shows communication with the bile duct. OLS plays an important role in distinguishing between MCN-L and IPNB. Thus, OLS has been established as a diagnostic criterion for MCN-L by the WHO.

MCN-L is rare, accounting for less than 5% of all cystic lesions of the liver [3]. Generally, MCN-L occurs predominantly in females, as in our patient. Typical symptoms of MCN-L are nonspecific, such as epigastralgia and abdominal distension [4]. MCN-L is sometimes asymptomatic and rarely presents with obstructive jaundice because it does not communicate with the bile duct. However, our case was symptomatic because of a polypoid lesion that grew into the bile duct and caused bile duct obstruction.

On imaging examinations such as ultrasonography, CT, and magnetic resonance imaging (MRI), MCN-L often forms a cyst-in-cyst structure without communication to the bile duct. It is a multilocular cyst with an internal septum, and its contents are mainly mucus. The sensitivity and specificity of imaging findings for diagnosing MCN-L have been reported to be 81% and 95%, respectively [5]. There is thickening of the cyst wall and a contrast effect with or without calcification. MRI findings changes depending on the composition of intracystic liquid. It is typically hypointense on T1-weighted images and hyperintense in T2-weighted images. However, it varies depending on the presence of hemorrhage or protein content [6].

It is difficult to differentiate between MCN-L and IPNB preoperatively. Another differential diagnosis is cholangiocarcinoma with cystic changes [7]. In our patient, it was difficult to preoperatively differentiate MCN-L from other diseases as although a cyst-in-cyst appearance was present, there was a nodule protruding into the bile duct, which was atypical for MCN-L.

The differential diagnoses of nodules formed in the bile ducts include hepatocellular carcinoma (HCC) with bile duct invasion and intraductal growth (IG) cholangiocellular carcinoma (CCC). When these tumors grow into the bile duct, the lesions in the bile duct have an irregular surface. Takano et al. stated that the smooth, round surface of the tumor in the bile duct is characteristic of MCN-L, unlike HCC invasion and IG-type CCC [8]. In our case, ERC revealed a smooth oval contrast defect similar to previously reported cases. Preoperative histopathological examination of biopsy for the nodular lesion in the bile duct also showed no malignant findings.

Histologically, MCN-L is a cyst-forming epithelial tumor composed of cuboidal or columnar epithelium and variable mucin-producing epithelium. The subepithelial layer consisted of densely organized, spindle-shaped cells resembling OLS. OLS is a mesenchyme consisting mainly of dense spindle-shaped cells and resembles the native ovarian interstitium not only in its histological morphology, but also in its immunohistochemical characteristics, including hormone receptor expression, such as ER and progesterone receptor expression. OLS in epithelial tissue forming cysts is necessary for the pathological diagnosis of MCN-L. The presence of gonads in the vicinity of the liver is thought to be responsible for the migration of gonadal cells to the liver surface during embryonic development and for the formation of the ovarian stroma in MCN-L [9]. In this case, OLS was observed not only in the area forming the cyst wall, but also in the polypoid nodule protruding into the bile duct. These findings confirmed that MCN-L had spread into the bile ducts.

For treatment, complete resection is recommended because MCN-L is considered a potential malignancy even though previous reports have suggested that the probability of malignant transformation is less than 20% [10]. Surgical resection of patients with MCN-L has been reported to achieve a 100% 5-year survival rate [4]. Therefore, even in asymptomatic cases of MCN-L, surgical treatment is recommended. Indication for liver resection includes a tumor size of the tumor larger than 100 mm, the tumor increases over time, or symptoms appear [2].

We searched previous reported cases of MCN-L which grew into the bile duct, using the key words “mucinous cystic neoplasm of the liver” on PubMed (Table 1) [8, 11–26]. We chose only MCN-L diagnoses based on the presence of OLS and excluded cases that were complicated by IPNB. The median age of the 19 patients, including our patient, was 41 years (range, 25–69 years). Our patient is the oldest among the patients and all patients were women. The median tumor size was 55 mm (range, 18–83 mm). Zen et al. reported that the mean tumor diameter in 54 MCN-L cases was 100 mm (29–240 mm) [2]. MCN-L prolapsing into the bile duct tends to be smaller size than normal MCN-L. This may be because MCN-L with growth into bile duct causes symptoms such as liver dysfunction and obstructive jaundice even if small tumor size. The most common primary site of the tumor was S4, similar to that in our patient. No malignant findings were observed in any of the reported cases. Prolapse of a nodular lesion into bile duct is not always associated with tumor malignancy.

**Table 1:** Review of reported cases of mucinous cystic neoplasm of the liver which grew into the bile duct

Case	Year	Author	Age (years)	Sex	Symptoms	Tumor size	Primary site of the tumor	Operation	Pathological diagnosis
1	2004	Shima	62	F	Jaundice	41 mm	CBD	Bile duct resection	Cystadenoma of the CBD
2	2004	Park	42	F	Jaundice	N/A	CBD	Bile duct resection	Biliary cystadenoma
3	2004	Preetha	58	F	Hypochondrial pain, jaundice	N/A	LHD, CBD	Left hemihepatectomy	Biliary cystadenoma
4	2006	Baudin	40	F	Epigastric pain, jaundice	70 mm	Left lobe	Left hemihepatectomy	Biliary cystadenoma
5	2009	Gonzalez	32	F	Abdominal pain, jaundice	79 mm	S3	Left hemihepatectomy	Biliary cystadenoma
6	2009	Siriwardana	25	F	Hypochondrial pain	55 mm	S4	Left hemihepatectomy, cholecystectomy	Biliary cystadenoma
7	2009	Yi	56	F	Hypochondrial pain, jaundice	55 mm	S4	Left hemihepatectomy	Biliary cystadenoma
8	2010	Saravanan	34	F	Jaundice	45 mm	S4	Bile duct resection	Biliary cystadenoma
9	2011	Hennessey	54	F	Abdominal pain	18 mm	CBD	Bile duct resection	Biliary cystadenoma
10	2011	Harmouch	57	F	Hypochondrial pain, jaundice	50 mm	S4	Left hemihepatectomy, bile duct resection, cholecystectomy	Hepatobiliary cystadenoma
11	2012	Vyas	41	F	Epigastric pain	30 mm	S4	Left hemihepatectomy, bile duct resection	Hepatobiliary cystadenoma
12	2012	Soochan	62	F	Dysuria	N/A	LHD	Extended left hemihepatectomy, bile duct resection	Extrahepatic cystadenoma
13	2012	Abe	28	F	Abdominal pain	73 mm	S4	Segmentectomy, bile duct resection, cholecystectomy	Hepatobiliary cystadenoma
14	2013	Rayapudi	37	F	Abdominal bloating	29 mm	S4	Left hemihepatectomy, bile duct resection	Biliary cystadenoma
15	2013	Chandrasinghe	39	F	Jaundice	N/A	S4	Left hemihepatectomy, bile duct resection	Biliary mucinous cystadenoma
16	2015	Takano	57	F	Abdominal pain, fever	83 mm	S4	Left hemihepatectomy, bile duct resection, cholecystectomy	MCN-L
17	2015	Takano	26	F	Jaundice	61 mm	S4	Extended left hemihepatectomy, bile duct resection	MCN-L
18	2018	Pattarapuntakul	27	F	Jaundice	56 mm	CBD	Left hemihepatectomy	MCN-L
19	2021	our case	69	F	Hypochondrial pain, jaundice	40 mm	S4	Left hemihepatectomy, bile duct resection	MCN-L

*F* female, *N/A* not applicable, *CBD* common bile duct, *LHD* left hepatic duct, *S* segment, *MCN-L* mucinous cystic neoplasm of the liver

MCN-L is usually considered a cystic tumor that does not communicate with the bile ducts, but it can grow into the bile ducts, as noted in our case. Although it is difficult to obtain a definitive diagnosis of MCN-L preoperatively, surgical treatment can provide a good prognosis and feasible treatment owing to its malignant potential. As the cause of MCN-L communication with the bile ducts remains still unknown, further investigation is needed on the accumulation of cases.

## Conclusion

Herein, we report an extremely rare case of MCN-L with a polypoid nodule prolapsing into the bile duct. Although MCN-L rarely communicates with the bile duct, we should keep in mind that MCN-L could grow into the bile duct, occasionally causing obstructive jaundice. Further case accumulation and pathophysiological analysis are needed. We believe that this case report contributes to the elucidation of its pathogenesis.

## Data Availability

Not applicable.

## Abbreviations

MCN-L: Mucinous cystic neoplasm of the liver
WHO: World Health Organization
IPNB: Intraductal papillary mucinous neoplasm of bile duct
OLS: Ovarian-like stroma
CT: Computed tomography
ERC: Endoscopic retrograde cholangiography
ER: Estrogen receptor
MRI: Magnetic resonance imaging
HCC: Hepatocellular carcinoma
CCC: Cholangiocellular carcinoma
